# Supplementary material for: A genome-wide analysis of YY1 and TFAP2 competition on overlapping motifs reveals their roles in HPV-induced carcinogenesis
Source: PLoS Pathog. 2025 Sep 15;21(9):e1013524. doi: 10.1371/journal.ppat.1013524 (PMC12445741; doi:10.1371/journal.ppat.1013524)
Supplement: S5 Table — (DOCX) [file ppat.1013524.s005.docx]

**Table S5**. Oligo information

| **Primer target and name** | **Position (hg19)** | **Sequence (5' to 3')*** | **Restriction enzyme site** | **Notes** |
| --- | --- | --- | --- | --- |
| **oJFX 07** | **chr15:89932642-89932660** | **ACAGCCACCAGCATCTCTC** |  | **lnc-FANCI-2 Taqman qPCR assay** |
| **lnc-FANCI-2 qPCR probe** | **chr15:89938268-89938294** | **TGAAGTGAAGTATGATCGGACAGCCTC** |  |  |
| **oJFX 08** | **chr15:89938333-89938312** | **CCACAATTCCTCTGTCTCCATT** |  |  |
| **siYY1 #1 sense** | **chr14:100706005-100706025** | **GACGACGACUACAUUGAACAATT** |  | **siYY1 #1** |
| **siYY1 #1 anti-sense** | **chr14:100706025-100706005** | **UUGUUCAAUGUAGUCGUCGUCTT** |  |  |
| **siYY1 #2 sense** | **chr14:100743853-100743873** | **CGAUGGUUGUAAUAAGAAGUUTT** |  | **siYY1 #2** |
| **siYY1 #2 anti-sense** | **chr14:100743873-100743853** | **AACUUCUUAUUACAACCAUCGTT** |  |  |
| **oHBL 44** | **chr15:89921752-89921775** | ***B-*GCGTTCGCGGGAGCCTGAGGCGCT** |  | **Oligo pull-down oligo1** |
| **oHBL 45** | **chr15:89921775-89921752** | **AGCGCCTCAGGCTCCCGCGAACGC** |  |  |
| **oHBL 46** | **chr15:89921935-89921958** | ***B-*CCTTTCCAAGATTCACACCTCGTC** |  | **Oligo pull-down oligo2** |
| **oHBL 47** | **chr15:89921958-89921935** | **GACGAGGTGTGAATCTTGGAAAGG** |  |  |
| **oHBL 50** | **chr15:89922169-89922192** | ***B-*GCCCGGGAGGCCATTTTGAGAGCG** |  | **Oligo pull-down oligo4** |
| **oHBL 51** | **chr15:89922192-89922169** | **CGCTCTCAAAATGGCCTCCCGGGC** |  |  |
| **oHBL 52** | **chr15:89922199-89922225** | ***B-*GGGGCGGCAAGATGGCTGCGTGGGCAC** |  | **Oligo pull-down oligo5** |
| **oHBL 53** | **chr15:89922225-89922199** | **GTGCCCACGCAGCCATCTTGCCGCCCC** |  |  |
| **oHBL 60** | **chr15:89922169-89922192** | ***B-*GCCCGGGAGGTCGATTTGAGAGCG** |  | **Oligo pull-down oligo4-M** |
| **oHBL 61** | **chr15:89922192-89922169** | **CGCTCTCAAATCGACCTCCCGGGC** |  |  |
| **oHBL 123** | **chr15:89922169-89922192** | **B-GCCCGGGACCGCATTTTGAGAGCG** |  | **Oligo pull-down oligo4-M1** |
| **oHBL 124** | **chr15:89922192-89922169** | **CGCTCTCAAAATGCGGTCCCGGGC** |  |  |
| **oHBL 125** | **chr15:89922169-89922192** | **B-GGGGGGGAGGCCATTTTGAGAGCG** |  | **Oligo pull-down oligo4-M2** |
| **oHBL 126** | **chr15:89922192-89922169** | **CGCTCTCAAAATGGCCTCCCCCCC** |  |  |
| **oHBL 87** | **chr15:89920048-89920069** | **CTGGAGGTGACAATTCCTGATT** |  | **ChIP: promoter**  **-2242 to -2062** |
| **oHBL 76** | **chr15:89920228-89920209** | **CACCG/TCAAGGATACAGAGTCGTAA** |  |  |
| **oHBL 14** | **chr15:89920982-89921001** | **GGAAGATCT/GGTGGCTGGAAGCGTGGCTA** | **Bgl II** | **ChIP: promoter**  **-1308 to -932** |
| **oHBL 06** | **chr15:89921358-89921334** | **AGCTGCTGGCGAGTTCGTGACTGCG** |  |  |
| **oHBL 27** | **chr15:89921982-89922001** | **AGTCGGGGTACC/TACGGCCTTCCGAACCCGAA** | **Kpn I** | **ChIP: promoter**  **-308 to +108** |
| **oHBL 07** | **chr15:89922398-89922375** | **GGCAGGAAGACGGCAGCCACTCTT** |  |  |
| **oHBL 83** | **chr15:89923385-89923404** | **CACCG/AACCTCCCAGTCTAAAGCCC** |  | **ChIP: promoter**  **+1095 to +1310** |
| **oHBL 90** | **chr15:89923600-89923578** | **ACATGAGCAGTTGACCTACATAA** |  |  |
| **oHBL 120** | **chr15:89927820-89927839** | **GCTGTGCCTCAGTCTGTAAA** |  | **ChIP: promoter**  **+5530 to +6058** |
| **oHBL 114** | **chr15:89928348-89928327** | **GTGTCCTGTGGGTTCTGAATTA** |  |  |
| **oYL01** | **chr17:25621115-25621138** | **B-CGGCGCCGCCCGCCATTTTGACTC** |  | **Oligo pull-down WSB1** |
| **oYL02** | **chr17:25621138-25621115** | **GAGTCAAAATGGCGGGCGGCGCCG** |  |  |
| **oYL03** | **chr17:62915553-62915579** | **B-GATCGCCTTCAGCCGCCATCTTGTTTC** |  | **Oligo pull-down LRRC37A3** |
| **oYL04** | **chr17:62915579-62915553** | **GAAACAAGATGGCGGCTGAAGGCGATC** |  |  |
| **oYL05** | **chr10:103911945-103911969** | **B-CTCCGCCCTTAACCAAGATGGCGAT** |  | **Oligo pull-down NOLC1** |
| **oYL06** | **chr10:103911969-103911945** | **ATCGCCATCTTGGTTAAGGGCGGAG** |  |  |
| **oYL07** | **chr12:77158038-77158061** | **B-AACACCAAGATGGCGGACGGCCCG** |  | **Oligo pull-down ZDHHC17** |
| **oYL08** | **chr12:77158061-77158038** | **CGGGCCGTCCGCCATCTTGGTGTT** |  |  |
| **oYL09** | **chr10:112327441-112327469** | **B-CGCCGCCATTTTGTTTGGCTGAGGGGAGC** |  | **Oligo pull-down SMC3** |
| **oYL10** | **chr10:112327469-112327441** | **GCTCCCCTCAGCCAAACAAAATGGCGGCG** |  |  |
| **oYL11** | **chr5:32444390-32444413** | **B-AGTTGCCGGTCGCCATTTTGGCAT** |  | **Oligo pull-down ZFR** |
| **oYL12** | **chr5:32444413-32444390** | **ATGCCAAAATGGCGACCGGCAACT** |  |  |
| **oYL13** | **chr19:15490124-15490151** | **B-CGCCCCCCTAGCGCCGCCATTTTGTGGC** |  | **Oligo pull-down AKAP8** |
| **oYL14** | **chr19:15490151-15490124** | **GCCACAAAATGGCGGCGCTAGGGGGGCG** |  |  |
| **oYL15** | **chr19:15529591-15529617** | **B-CGGCGCCATTTTGTGACCGCAGGGAAG** |  | **Oligo pull-down AKAP8L** |
| **oYL16** | **chr19:15529617-15529591** | **CTTCCCTGCGGTCACAAAATGGCGCCG** |  |  |
| **oYL17** | **chrX:70503439-70503465** | **B-GAACGCCATTTTGTACCCCTTGGCAGG** |  | **Oligo pull-down NONO** |
| **oYL18** | **chrX:70503465-70503439** | **CCTGCCAAGGGGTACAAAATGGCGTTC** |  |  |
| **oYL19** | **chr11:118938518-118938542** | **B-GGGAGCCCTGGGCCAAAATGGCGGC** |  | **Oligo pull-down VPS11** |
| **oYL20** | **chr11:118938542-118938518** | **GCCGCCATTTTGGCCCAGGGCTCCC** |  |  |
| **oYL21** | **chr2:38604387-38604410** | **B-CGTCCCCCTCCGCCATCTTGTACC** |  | **Oligo pull-down ATL2** |
| **oYL22** | **chr2:38604410-38604387** | **GGTACAAGATGGCGGAGGGGGACG** |  |  |
| **oYL23** | **chr2:242576959-242576983** | **B-CCCGGCCGTACGCCAAAATGGCGGC** |  | **Oligo pull-down ATG4B** |
| **oYL24** | **chr2:242576983-242576959** | **GCCGCCATTTTGGCGTACGGCCGGG** |  |  |
| **oYL25** | **chr6:5260947-5260974** | **B-GCGCGACTGGAGGCTGCCATTTTGGAAA** |  | **Oligo pull-down LYRM4** |
| **oYL26** | **chr6:5260974-5260947** | **TTTCCAAAATGGCAGCCTCCAGTCGCGC** |  |  |
| **oYL27** | **chr6:15245699-15245722** | **B-CCGTGGCCATAGCCATTTTGTAGT** |  | **Oligo pull-down JARID2** |
| **oYL28** | **chr6:15245722-15245699** | **ACTACAAAATGGCTATGGCCACGG** |  |  |
| **oYL29** | **chr6:64283337-64283360** | **B-TCAGACAAAATGGCCTCGGCGCCC** |  | **Oligo pull-down PTP4A1** |
| **oYL30** | **chr6:64283360-64283337** | **GGGCGCCGAGGCCATTTTGTCTGA** |  |  |
| **oYL31** | **chr16:58663725-58663751** | **B-CTCTACAAAATGGCGCCGGAGGTCGCG** |  | **Oligo pull-down CNOT1** |
| **oYL32** | **chr16:58663751-58663725** | **CGCGACCTCCGGCGCCATTTTGTAGAG** |  |  |
| **oYL33** | **chr1:204380935-204380959** | **B-AAGCCCAAAATGGCCGCAGGGCCCG** |  | **Oligo pull-down PPP1R15B** |
| **oYL34** | **chr1:204380959-204380935** | **CGGGCCCTGCGGCCATTTTGGGCTT** |  |  |
| **oYL35** | **chr16:57481409-57481432** | **B-GCTTCCAAAATGGCGGCGGCGGCG** |  | **Oligo pull-down CIAPIN1** |
| **oYL36** | **chr16:57481432-57481409** | **CGCCGCCGCCGCCATTTTGGAAGC** |  |  |
| **oYL37** | **chr6:33290931-33290955** | **B-GGCCGCCATTTTGCCGTACGGCACT** |  | **Oligo pull-down DAXX** |
| **oYL38** | **chr6:33290955-33290931** | **AGTGCCGTACGGCAAAATGGCGGCC** |  |  |
| **oYL39** | **chr6:150039183-150039206** | **B-CCGCCGCCGCCGCCATTTTGCCTT** |  | **Oligo pull-down LATS1** |
| **oYL40** | **chr6:150039206-150039183** | **AAGGCAAAATGGCGGCGGCGGCGG** |  |  |
| **oYL41** | **chr1:204378701-204378681** | **GAGCCAAGAAAGTGAATGTCC** |  | **PPP1R15B qPCR assay** |
| **oYL42** | **chr1:204375378-204375396** | **ATGGTCCTTTGCGATCCTC** |  |  |
| **oYL43** | **chr17:62856465-62856444** | **CAGGTGAGAGACAGATGGAAAG** |  | **LRRC37A3 qPCR assay** |
| **oYL44** | **chr17:62856358-62856380** | **TCTGGAATGTACGATGGGTTTAG** |  |  |
| **oYL45** | **chr12:6645933-6645954** | **AAATCCCATCACCATCTTCCAG** |  | **GAPDH qPCR assay** |
| **oYL46** | **chr12:6646798-6646778** | **AGGGGCCATCCACAGTCTTCT** |  |  |

Note, *, Mutated nucleotide(s) are underlined. Unrelated sequence and gene specific sequence or introduced deletion are separated by "/ ". B stands for biotin.
