# Supplementary figures and images for: A genome-wide analysis of YY1 and TFAP2 competition on overlapping motifs reveals their roles in HPV-induced carcinogenesis
Source: PLoS Pathog. 2025 Sep 15;21(9):e1013524. doi: 10.1371/journal.ppat.1013524 (PMC12445741; doi:10.1371/journal.ppat.1013524)

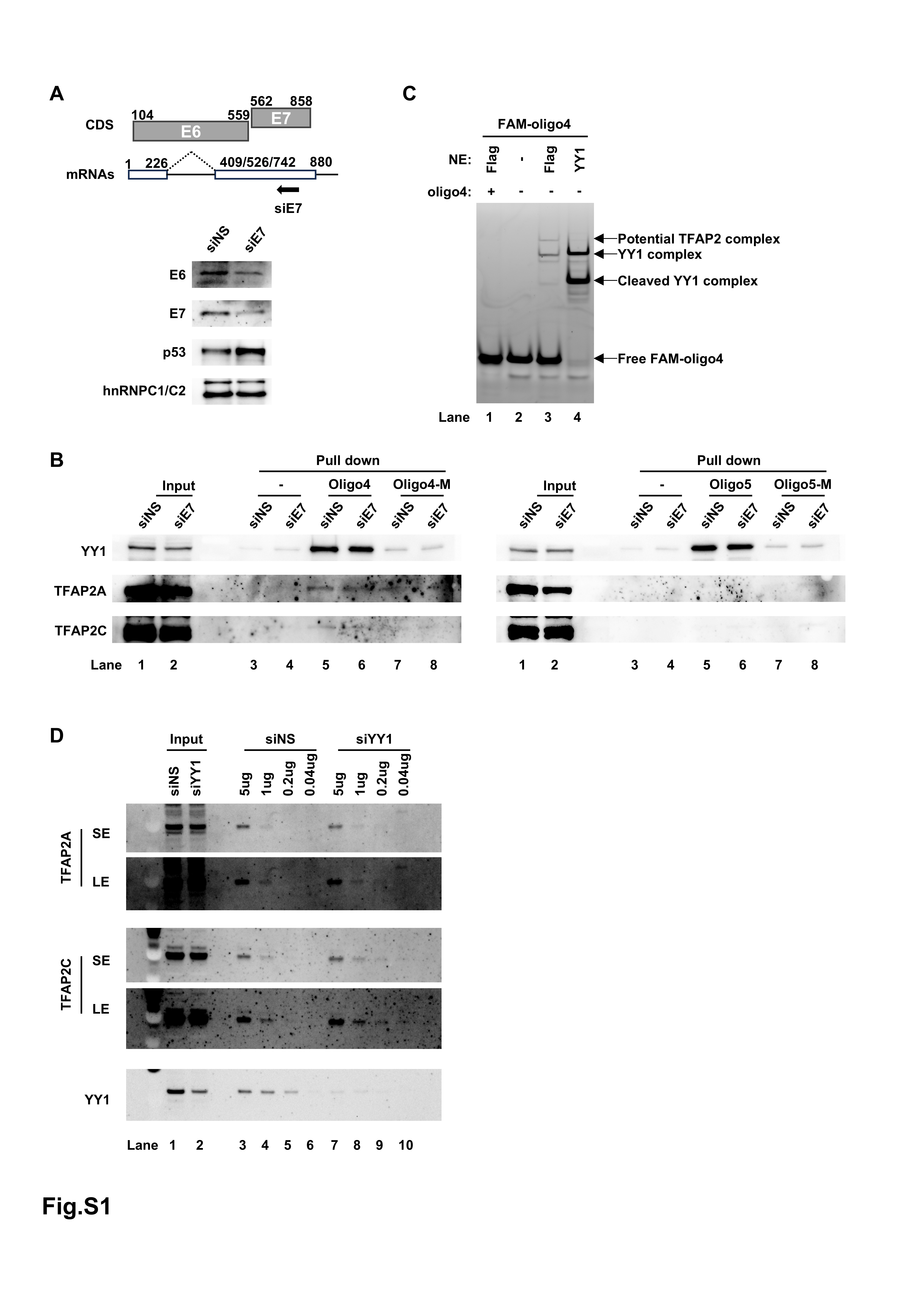

Supplement: S1 Fig — Soluble nuclear extracts were prepared from E7-siRNA transfected CaSki cells, and the expression levels of HPV16 E6, E7, and p53 were evaluated by western blot using anti-E6 (#GTX132686, GeneTex), anti-E7 (#GTX637546, GeneTex), and anti-p53 (#10442–1-AP, Proteintech) antibodies. hnRNPC1/C2 (#68447–1-Ig, proteintech) was used as an internal control. (B) Binding of TFAP2A and TFAP2C to the YY1 motif B. Pulldowns were performed using oligo4, oligo4-M, oligo5, and oligo5-M with fractionated nuclear extracts isolated from HeLa cells with the indicated siRNA knockdown. (C) Validation of TFAP2 and YY1 competition for oligo4 by EMSA. FAM-labeled oligo4 was synthesized by Sangon Biotech, and unlabeled oligo4 was used as a competitor. Nuclear extracts containing YY1-Flag (YY1) or Flag-control (Flag) were prepared as described in Fig 2E. Binding reactions were performed by incubating 0.1 pmol of FAM-labeled oligo4 with the indicated nuclear extracts in 1 × EMSA/Gel-Shift binding buffer (#GS005, Beyotime Biotech) for 20 minutes at room temperature. Samples were then resolved on a 4–20% BeyoGel TBE PAGE gel (#D0185S, Beyotime Biotech). The gel was scanned using an Amersham Typhoon imager (GE Healthcare). Lane 1 contained 10 pmol of unlabeled oligo4 as a competitor. NE: nuclear extract. (D) Validation of TFAP2 and YY1 competition via oligonucleotide titration assay. Soluble nuclear extracts were prepared from CaSki cells treated with non-specific siRNA (siNS) or YY1 siRNA (siYY1). LE, long exposure; SE, short exposure. (TIF) [file ppat.1013524.s006.tif]

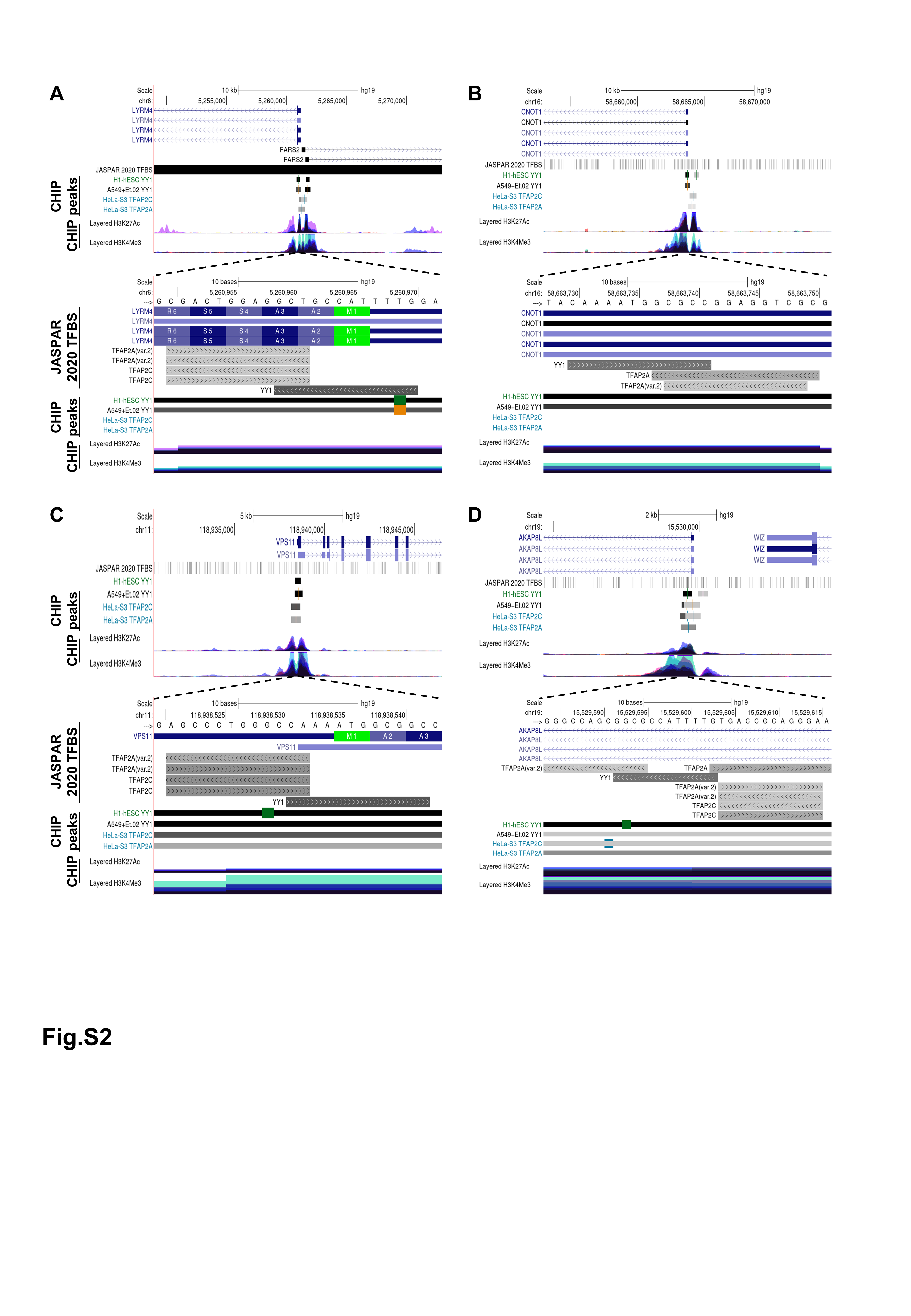

Supplement: S2 Fig — The upper panel views in each panel show the genome locus of each gene (GRCh37/hg19) and the lower panel views in each panel are zoomed in the YY1 and TFAP2 binding motifs with all regulation tracks. (TIF) [file ppat.1013524.s007.TIF]

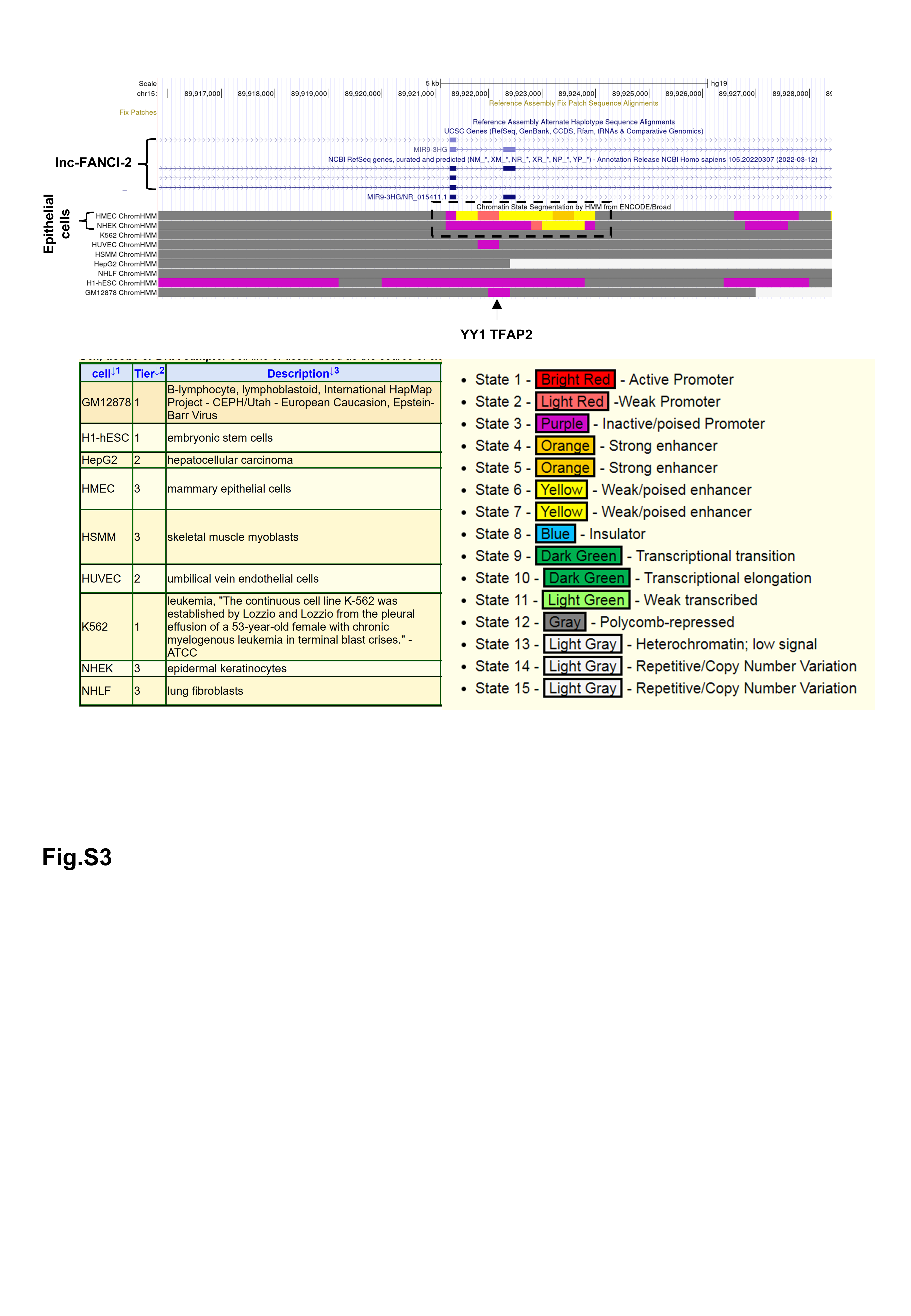

Supplement: S3 Fig — (TIF) [file ppat.1013524.s008.TIF]
