# Supplementary material for: A genome-wide analysis of YY1 and TFAP2 competition on overlapping motifs reveals their roles in HPV-induced carcinogenesis
Source: PLoS Pathog. 2025 Sep 15;21(9):e1013524. doi: 10.1371/journal.ppat.1013524 (PMC12445741; doi:10.1371/journal.ppat.1013524)
Supplement: Attachment — Submitted filename: Review comments on PPATHOGENS-D-25-00313.docx [file ppat.1013524.s009.docx]

Comments on "A genome-wide analysis of YY1 and TFAP2 competition on overlapping motifs reveals their roles in HPV-induced carcinogenesis" by Liu et al.

This report investigates the regulatory mechanism of the long non-coding RNA lnc-FANCI-2 in HPV-driven cervical carcinogenesis. The report provides novel insights into the antagonistic roles of YY1 and TFAP2 in regulating lnc-FANCI-2 and broader transcriptional networks, supported by omics validation. The development of a DNA oligo pulldown method to map competitive TF binding is technically innovative and broadly applicable.

Overall, this manuscript presents valuable findings about the transcriptional regulation of lnc-FANCI-2 in the context of HPV infection and proposes a new model of transcription factor competition. However, the manuscript would benefit from clearer logic and more precise language to improve readability and understanding. With major adjustments, I recommend this article be accepted for publication.

Major Points:

1. In the Introduction, the authors describe in separate paragraphs that the E6 and E7 genes are crucial for maintaining the transformed state in HPV-induced cancer cells, and that the YY1 gene is upregulated in HPV-positive cancer cells, activating cancer-associated lnc-FANCI-2. However, the lack of a clear connection between the discussion of E6 and E7 in the first paragraph and the upregulation of YY1 in the second paragraph makes it difficult to understand the background and significance of the research. It is suggested to include a discussion on how E7 regulates YY1, leading to the research objective of this study, in order to improve the logical flow of the writing.

Figure S1 and Figure 1 aim to identify the regulatory or binding proteins of the lnc-FANCI-2 core promoter. However, it is unclear why E7 knockdown was included in the DNA oligo pulldown-coupled mass spectrometry experiment. The knockdown of E7 does not appear to be directly related to the experimental objective described in this section. The authors should provide a clearer explanation of the experimental rationale behind the use of E7 knockdown.

2. The conclusion that TFAP2 competes with YY1 for binding to the YY1-B motif seems unconvincing. For example, in Fig. S2D, YY1 enrichment in CaSki cells (Oligo4) is significantly higher compared to HFK cells, but TFAP2A enrichment does not decrease; rather, it increases. Moreover, in Fig. S2E, when TFAP2A is knocked down in CaSki cells, one would expect YY1 binding to increase, and lnc-FANCI-2 expression to rise. In Fig. S2A-B, TFAP2 bound to the lnc-FANCI-2 promoter similarly in HFKs and CaSki cells, which raises doubts about the competition model. Therefore, the conclusion that TFAP2 competes with YY1 for binding requires additional experimental evidence.
3. The authors developed a DNA oligo pull-down method to compare binding affinities or determine mutually exclusive binding (Fig. 1). However, the reliability of this new method is not further evaluated. It is recommended to include a positive control group or incorporate additional methods for comparative analysis to strengthen the validity of the findings.

Minor Points:

1. The writing needs to be improved to enhance the readability and logical flow of the manuscript. For example, the first two sentences of the Results section, 'YY1-B is more critical for the lnc-FANCI-2 promoter activity. To further identify the regulatory or binding proteins of the lnc-FANCI-2 core promoter, we performed DNA oligo pulldown-coupled mass spectrometry using soluble nuclear extract (Nuc) from HPV16+ CaSki cells with or without E7 knockdown,' are not clearly connected. The statement about YY1-B does not logically lead to the description of the experiment in the second sentence.
2. The insertion of a working model in Figure 2 is not ideal for the structure of the manuscript. It would be better to introduce the relationships between YY1, TFAP2, E7, lnc-FANCI-2, and miR-29a in the Introduction and use Fig. 2 as an abstract diagram for clarity.
3. In several Western blot experiments, p53 expression is measured (e.g., Fig. S1B, where E7 siRNA targeting both E6 and E7 transcripts resulted in the accumulation of nuclear p53). However, the role of p53 is not explained, and the connection between this result and the main theme of the article is unclear. Further clarification is needed on why p53 was measured and how these results tie into the overall hypothesis.
4. Since both YY1 and TFAP2 are transcription factors, their overlapping binding motifs likely affect the expression of numerous genes. In Figure 3B, the statement that "these CBS potentially regulate cell junction assembly, cell-cell adhesion via plasma membrane adhesion molecules, and keratinocyte proliferation" seems speculative without detailed reasoning or evidence linking these pathways directly to the mechanisms of HPV-induced carcinogenesis. The experiment design of genome-wide screening of YY1 and TFAP2 competitive binding sites to find genes related to HPV-driven carcinogenesis seems inadequately substantiated.
5. Fig. 4A–B and Fig. S3 mention H3K27ac and H3K4Me3 marks in the promoter regions, but the manuscript briefly mentions these findings without explaining their significance or the impact they have on the study’s conclusions. More detail on these marks and their relevance to the research would be beneficial.
6. In Fig. 4G–H, the authors demonstrate that high levels of either PPP1R15B or LRRC37A correlate with a worse survival rate in cervical cancer patients. This experiment effectively supports the relevance of YY1 and TFAP2 competitive binding sites in cervical cancer and ties well into the manuscript’s central theme.
7. In the Discussion, the authors mention that "the residual TFAP2 binding does not repress transcription." This point is unclear and warrants further experimental verification or more in-depth discussion to clarify its implications.
