# Supplementary material for: A genome-wide analysis of YY1 and TFAP2 competition on overlapping motifs reveals their roles in HPV-induced carcinogenesis
Source: PLoS Pathog. 2025 Sep 15;21(9):e1013524. doi: 10.1371/journal.ppat.1013524 (PMC12445741; doi:10.1371/journal.ppat.1013524)
Supplement: Attachment — Submitted filename: revision-2.docx [file ppat.1013524.s010.docx]

**Reviewers' Comments:**

Reviewer's Responses to Questions

# Part I - Summary

Please use this section to discuss strengths/weaknesses of study, novelty/significance, general execution and scholarship.

Reviewer #1: In general, this is an interesting study in which they discovering new TFAP2 family members that compete with YY1 for binding at overlapping sites of the lnc-FANCI-2 promoter. Basically, in primary epithelial cells, TFAP2 binding led to lnc-FANCI-2 silencing. In contrast, in HPV-positive cancer cells, increased YY1 levels displaced TFAP2, alleviating repression. They also did a genome-wide predictions and identified thousands of YY1 and TFAP2 competition binding sites (CBSs), many overlapping with CHIP peaks for YY1, TFAP2A, and TFAP2C, predominantly in promoter regions. Even though these interesting findings, there are a few questions about presented data need to be addressed.

Reviewer #2: Comments on "A genome-wide analysis of YY1 and TFAP2 competition on overlapping motifs reveals their roles in HPV-induced carcinogenesis" by Liu et al.

This report investigates the regulatory mechanism of the long non-coding RNA lnc-FANCI-2 in HPV-driven cervical carcinogenesis. The report provides novel insights into the antagonistic roles of YY1 and TFAP2 in regulating lnc-FANCI-2 and broader transcriptional networks, supported by omics validation. The development of a DNA oligo pulldown method to map competitive TF binding is technically innovative and broadly applicable.

Overall, this manuscript presents valuable findings about the transcriptional regulation of lnc-FANCI-2 in the context of HPV infection and proposes a new model of transcription factor competition. However, the manuscript would benefit from clearer logic and more precise language to improve readability and understanding.

Reviewer #3: This is a follow-on study of the YY1-dependent transcriptional regulation of lnc-FANCI-2 transcription in HPV-driven cervical cancer. The authors identify several novel interacting proteins of the YY1 binding region of the lnc-FANCI-2 promoter using in vitro oligo pull down assays. The findings are interesting. However, they suggest that while HPVE7 is required for the binding of YY1 to the lnc-FANCI-2 promoter, that a competitive binder to the same region, TFAP2, is not affected by E7 expression levels. The data underpinning these findings are a little weak (and mostly buried in the supplemental figures). I do feel that for publication in PLoS Pathogens, the viral, host-pathogen interaction aspects of this study should be enhanced before it can be considered.

Some specific comments are listed below:

The YY1 binding sites in the lnc-FANCI-2 promoter need to be better introduced in the text – the results start with a statement about YY1-B – this is not easy to follow without an initial definition of YY1-A/B and their function.

Response: We appreciate the reviewer’s suggestion for clearer context about YY1-A/B binding sites in the lnc-FANCI-2 promoter. In the revised manuscript, we have expanded the introduction to define these elements and their functional significance: Introductin: Lines 49-50, “HPV16 E7 enhances lnc-FANCI-2 expression by interacting with YY1. Two essential YY1 binding motifs upstream of lnc-FANCI-2's transcription start site are crucial for its transcriptional activity (Fig.1A), with YY1 knockdown or motif deletion causing a 70–80% reduction in lnc-FANCI-2 expression in cervical cancer cells.” Results: Lines 57-59, “Although two YY1-binding motifs, YY1-A and YY1-B (Fig. 1A), in the lnc-FANCI-2 promoter region have very similar binding affinities to YY1, the YY1-B is more critical for the lnc-FANCI-2 promoter activity.”

Of the 28 proteins identified as specifically associated with the YY1-B motif, 12 of these proteins with ‘related to E7 KD-induced loss of binding’. It isn’t clear what these proteins are and what criteria have been used to assess dependence on E7 for binding; there are 13 proteins listed in table S1 that appear to be lost when E7 is depleted.

Response: We thank the reviewer for catching this issue. Line 68, The revised manuscript now states that 13 proteins (not 12) lost detectable binding to the YY1-B motif following E7 knockdown as evidenced by the absence of the identifying peptides in mass spectrometry analyses (Table S1).

A western blot showing the efficiency of E6 and E7 depletion with siRNA is required. Throughout the manuscript, the siRNA is described as E7-specific but it could affect both E6 and E7. The only readout for the efficacy for this siRNA is nuclear p53 (fig S1B) but this is not a readout of E7 levels. It is misleading that the authors state that the binding of particular proteins to the oligo is E7-specific when E6 and/or E7 could be involved.

Response: We appreciate the reviewer’s comment. As requested, we have included a Western blot (Fig. S1) demonstrating the efficiency of E6 and E7 depletion with the E7-targeting siRNA in CaSki cells. While the siRNA was designed against E7, we acknowledge that it also reduces E6 levels, as previously reported [1]. In the revised manuscript, we clarify this point (Lines 67–71) and avoid attributing effects solely to E7 depletion where E6 may also play a role. Lines 67-71: “Previous work demonstrated E7 siRNA also targeted E6 transcripts and resulted in the accumulation of nuclear p53 (Fig.1B and Fig.S1A). This effect was used as an indicator of effective E6/E7 knockdown. We identified 28 proteins in specific association with oligo 4 over the mutant oligo 4-M (>3 peptides with >2-fold changes), 13 of them related to knockdown-induced loss of binding (Table S1 and Fig.1C). “

FigS1E appears to show that knockdown of E7 enhances YY1 binding to oligo 5. This is at odds with the authors’ previous manuscript showing that knockdown of E7 reduces YY1 binding to the lnc-FANC-2 promoter region (at YY1 sites A and B). Please can the authors explain.

Response: We acknowledge the apparent discrepancy between the observed enhancement of YY1 binding to oligo 5 upon E7 knockdown (FigS1E) and our previous findings showing that E7 depletion reduces YY1 occupancy at the lnc-FANC-2 promoter by CHIP assay (YY1 sites A and B). While the in vitro pull-down assay suggests that E7 may reduce YY1’s intrinsic binding affinity to its target DNA motif, the ChIP data reflect a more complex in vivo regulatory mechanism.

We speculate that E7 may facilitate YY1 recruitment to chromatin potentially through interactions with transcriptional cofactors, chromatin remodelers that enhance YY1’s promoter occupancy despite its slightly weaker DNA-binding affinity in the presence of E7. This would explain why E7 knockdown reduces YY1 enrichment at the lnc-FANC-2promoter in vivo (as seen in ChIP) while increasing its binding to naked DNA in vitro. Further studies are needed to identify the specific mechanisms by which E7 modulates YY1’s chromatin association and transcriptional activity.

What does a motif score of 384 mean? Without some context of the scale used for these scores, and how this number is derived, it is meaningless.

Response: We appreciate the reviewer’s request for clarification regarding the TFAP2 motif score. The TFAP2 motif score of 384 was calculated using the JASPAR Position Weight Matrix (MA0810.1) , where higher scores indicate stronger predicted binding affinity. This score represents the log-odds ratio of the sequence matching TFAP2's binding preference, with scores above 300 correlating with strong binding in our experimental validation (oligo pull-down assays). We have revised the manuscript to clarify that this score is calculated from JASPAR database [2].

Fig 1B – the ‘reduced or lost’ binding of TRAF2C to the mutant oligos needs to be quanitifed. An experiment similar to those shown in Fig1D, comparing the WT and mutant oligos, would be helpful for this purpose.

Response: Thanks for the reviewer’s suggestion. In response, we have added a line graph to quantify the reduced or lost binding of TRAF2C in now Fig. 2B. This new panel provides a direct comparison of TRAF2C binding affinity, reinforcing our conclusion that the mutations significantly impair factors and DNA binding.

Figure S2B needs a loading control.

Response: We thank the reviewer for this important suggestion. To address the concern, the original membrane was stripped and reprobed using a monoclonal anti-SRSF3 antibody (#NBP2-76892, NOVUS, Littleton, CO) as following, which serves as a nuclear loading control. As noted in the figure legend, the pulldown inputs are nuclear extracts. The updated data of the loading control have been incorporated into the revised version of the manuscript.

The ChIP results in Fig S2A should be shown as bar charts – the data points are not contiguous and therefore a fitted line graph is not appropriate. The % of input in these graphs is add – reaching nearly 120% for TFAP2C in CaSki cells for example. ChIPs are notoriously inefficient and IP’ing 100% of input is very unlikely.

Response: We sincerely appreciate the reviewer's insightful feedback regarding the presentation and interpretation of our ChIP data in Fig S2A (now Fig.3). As recommended, we have replaced the fitted line graphs with bar charts to better represent the discrete nature of our data points.

Upon re-examination, we identified an error in our initial % input calculations. Specifically, we realized that while we compared ChIP intensity to input intensity directly from gel images, we failed to account for volume differences in the PCR setup: Only 0.5 μL (1%) of input DNA (from 50 μL chromatin prep) was used for PCR; In contrast, 5 μL (10× more) of ChIP DNA (from same 50 μL prep) was used. This oversight artificially inflated our % input values. We have now corrected the normalization by applying a 10× dilution factor to the ChIP samples to properly reflect the actual input percentages. The corrected analysis now shows % input values that align with expected ChIP efficiencies. For example, the TFAP2C value in CaSki cells has been adjusted from ~120% to ~12%, which is biologically plausible. In addition, we added “Input (10%) “ labels in the figure too.

What are the sequences of the, presumably control, oligos 1 and 2 used in the in vitro pull down experiment in Fig 3C? How were these chosen? What cells were used for the nuclear extracts used in these experiments?

Response: The oligo sequences, oligos 1 and 2 used in our pull-down assays were reused from our previous characterization of transcription factor binding sites in the lnc-FANCI-2 promoter [3]. We use oligo 1 (TFAP2-specific), oligo 5 (YY1-specific), and oligo 4 (dual TFAP2/YY1 binding) as positive controls, while oligo 2 (containing other factor binding sites) served as a negative control (sequences and genomic positions in Table S5; validation data in Fig. S2C-D, now Fig. 3C-D). All experiments used nuclear extracts from CaSki cells (HPV16+ cervical cancer line), as these cells endogenously express the transcription factors.

The ‘competitive binding’ shown in Figs 4C and D need to be quantified.

Response: We appreciate the reviewer's suggestion to quantify the competitive binding data. As shown in our new Fig.4D and E lower panels, quantitative measurements of protein binding percentages have been done. These quantifications confirm that TFAP2A and TFAP2C showed reduced binding while increased exogenous YY1 was used in the pull-down assays.

How was significance calculated in Fig4F – the differences look marginal.

Response: We thank the reviewer for raising this important question regarding our statistical analysis. The data represent six samples from two independent experiments. The gene expressions of each sample were analyzed in technical triplicate by qPCR (data not shown). The bar graph in Fig.4F shows average and standard deviation (SD) of the six samples, and P value was caculated using two-tailed Student's t-tests comparing each condition to siNS controls.

| PPP1R15B | EX1-sample1 | EX1-sample2 | EX1-sample3 | EX2-sample4 | EX2-sample5 | EX2-sample6 | AVE | SD | P value |
| --- | --- | --- | --- | --- | --- | --- | --- | --- | --- |
| siNS | 1.00132 | 1.00000 | 1.10000 | 0.95734 | 1.02705 | 1.00000 | 1.01429 | 0.0476 |  |
| siYY1#1 | 1.13184 | 1.15016 | 1.37695 | 0.94436 | 1.42937 | 1.43364 | 1.24439 | 0.199584 | 0.020584 |
| siYY1#2 | 1.41600 | 1.55036 | 1.48475 | 1.65515 | 1.63524 | 1.41561 | 1.52618 | 0.105065 | 7.36E-07 |
|  |  |  |  |  |  |  |  |  |  |
| LRRC37A3 | EX1-sample1 | EX1-sample2 | EX1-sample3 | EX2-sample4 | EX2-sample5 | EX2-sample6 | AVE | SD | P value |
| siNS | 0.73954 | 1.00000 | 1.02304 | 0.98924 | 0.91152 | 0.88993 | 0.92555 | 0.105041 |  |
| siYY1#1 | 1.45999 | 1.48665 | 1.57830 | 1.16054 | 1.41484 | 1.45999 | 1.42672 | 0.141262 | 3.84E-05 |
| siYY1#2 | 1.08269 | 1.39364 | 1.11336 | 1.15464 | 0.92180 | 1.02287 | 1.11484 | 0.158791 | 0.035129 |

# Part II – Major Issues: Key Experiments Required for Acceptance

Please use this section to detail the key new experiments or modifications of existing experiments that should be absolutely required to validate study conclusions.

Generally, there should be no more than 3 such required experiments or major modifications for a "Major Revision" recommendation. If more than 3 experiments are necessary to validate the study conclusions, then you are encouraged to recommend "Reject".

Reviewer #1: 1. In Figure S1D and S1E, better to provide TFAP2A data as well, making them consistent with other data. They also need to repeat these experiments (S1D and S1E) in other HPV+ cervical cancer cell lines such as HeLa to make sure: 1) whether it is cell line specific? 2) whether it is HPV 16 specific?

Response: We sincerely appreciate the reviewer’s suggestion. First, the original membranes were stripped and reprobed with anti-TFAP2A antibody (upper panels of the following pictures). Addtionally, we repeated the pull-down assay in CaSki cells (lower panels), which yielded consistent results. The reprobed TFAP2A data for oligo5 pulldown has been added to the revised figure. For oligo4 pulldown, the reprobed TFAP2A signal showed high background interference. Thus, we replaced it with the cleaner data from the newly performed pull-down experiment.

Additionally, we performed oligo pulldown assays using HeLa nuclear extracts, and the results were included in Fig. S1B. Overall, the data are consistent.

2. In Figure 3C, they described “all 20 CBS-derived oligos tested were YY1 binders”, what about TFAP2A and TFAP2C? It seems that they were also TFAP2A and TFAP2C binders?? Also, did they find some CBS which are only TFAP2A and TFAP2C dependent but not YY1 (as they found in the promoter of lnc-FANCI-2).

Response: We appreciate the reviewer's questions about TFAP2 binding specificity. Our genome-wide screening identified all CBs based on high stringent criteria (JASPAR motif score >520 and within ChIP-seq peaks), while TFAP2 binding was selected under less stringent conditions. Only oligos 1 and 4, and oligos from LRRC37A3, LYRM4, CNOT1, PPP1R15B, AKAP8L, and VPS11 promoter were validated as TFAP2 binders. The observed nonspecific TFAP2 interactions with other oligos likely reflect low-affinity binding to double-stranded DNA. Under these screening conditions, all identified CBS were YY1-dependent. We have clarified these experimental criteria and binding thresholds in materials and methods section in the supporting information.

3. In Figure 4, based on results from 4F it seems that YY1 is a repressor for these targeted genes? (which is different from its role in lnc-FANCI-2). Also, it is confusing that “high level of YY1 in HPV+ cancer cells” while “high levels of either PPP1R15B or LRRC37A correlated with a worse survival rate in patients with cervical cancer”. Any contradiction?

Response: We sincerely thank the reviewer for raising this important question about YY1's context-dependent functions. The observed regulatory differences highlight YY1's well-documented dual role as both a transcriptional activator and repressor [4,5]. While our data show YY1 activates lnc-FANCI-2 expression in cervical cancer cells, it simultaneously represses other targets including PPP1R15B and LRRC37A (Fig 4F). The correlation between elevated PPP1R15B/LRRC37A levels and poor prognosis suggests these genes may exhibit oncogenic properties when their YY1-mediated repression is relieved. This reveals YY1's complex involvement in cervical cancer pathogenesis, where it appears to: (1) directly activate the tumor suppression RNA lnc-FANCI-2, while (2) suppress other oncogenic pathways through repression of genes like PPP1R15B/LRRC37A. The precise mechanisms underlying these differential regulatory effects warrant further investigation in future studies.

Reviewer #2: 1. In the Introduction, the authors describe in separate paragraphs that the E6 and E7 genes are crucial for maintaining the transformed state in HPV-induced cancer cells, and that the YY1 gene is upregulated in HPV-positive cancer cells, activating cancer-associated lnc-FANCI-2. However, the lack of a clear connection between the discussion of E6 and E7 in the first paragraph and the upregulation of YY1 in the second paragraph makes it difficult to understand the background and significance of the research. It is suggested to include a discussion on how E7 regulates YY1, leading to the research objective of this study, in order to improve the logical flow of the writing.

Response: We sincerely appreciate the reviewer’s constructive suggestion to improve the logical flow between HPV oncoproteins and YY1-mediated transcriptional regulation. In the revised manuscript, we have connected E7’s role to YY1 regulation by adding a statement about E7’s functional interaction with YY1 in the second paragraph (Lines 38-39, “Notably, HPV16 E7 has been shown to functionally interact with the transcriptional regulator YY1, modulating its activity at specific promoters”), and the mechanistic link (E7→miR-29a suppression→YY1 upregulation) in the third paragraph (Lines 46-50, “It is upregulated due to reduced miR-29a in HPV-positive cancer cells, leading to the activation of the cancer-associated long non-coding RNA lnc-FANCI-2. Two essential YY1 binding motifs upstream of lnc-FANCI-2's transcription start site are crucial for its transcriptional activity16 (Fig.1A). YY1 knockdown or motif deletion20 causing a 70–80% reduction in lnc-FANCI-2 expression in cervical cancer cells.”). These changes mat better establish how E7’s manipulation of YY1 contributes to HPV-induced transcriptional reprogramming.

Figure S1 and Figure 1 aim to identify the regulatory or binding proteins of the lnc-FANCI-2 core promoter. However, it is unclear why E7 knockdown was included in the DNA oligo pulldown-coupled mass spectrometry experiment. The knockdown of E7 does not appear to be directly related to the experimental objective described in this section. The authors should provide a clearer explanation of the experimental rationale behind the use of E7 knockdown.

Response: We appreciate the reviewer’s request for clarification. We have clarified this rationale in the revised Results section to better emphasize how this experimental design addresses both HPV-dependent and independent regulatory mechanisms at the lnc-FANCI-2 promoter. Lines 59-64, “To comprehensively characterize how HPV16 E7 modulates the transcriptional complex at this locus, we performed DNA oligo pull-down-coupled mass spectrometry using soluble nuclear extract (Nuc) from HPV16+ CaSki cells with or without E7 siRNA transfection (Fig.1A and B). This experimental design allowed us to identify baseline protein interactions at the lnc-FANCI-2 promoter core region and distinguish E7-dependent binding partners through comparative analysis.”

2. The conclusion that TFAP2 competes with YY1 for binding to the YY1-B motif seems unconvincing. For example, in Fig. S2D, YY1 enrichment in CaSki cells (Oligo4) is significantly higher compared to HFK cells, but TFAP2A enrichment does not decrease; rather, it increases. Moreover, in Fig. S2E, when TFAP2A is knocked down in CaSki cells, one would expect YY1 binding to increase, and lnc- FANCI-2 expression to rise. In Fig. S2A-B, TFAP2 bound to the lnc-FANCI-2 promoter similarly in HFKs and CaSki cells, which raises doubts about the competition model. Therefore, the conclusion that TFAP2 competes with YY1 for binding requires additional experimental evidence.

Response: We appreciate the reviewer's thoughtful critique of our competition model and have conducted additional experiments to address these concerns. The competitive binding dynamics are highly context-dependent, and we present a more comprehensive explanation here:

Standard oligo pull-down experimental conditions (5μg oligos) show no competition due to sufficient DNA binding sites for all three factors (Fig. 2D, lane 2), we observe clear competitive displacement when either heavily reducing oligo amounts (Fig. 2D, lanes 3-7) or heavily increasing YY1 levels through exogenous expression in this in-vitro system (when large amount DNA were used) (Fig. 2E). As the standard experimental conditions were used in Fig. S2D (5 μg oligos per reaction), competition between TFAP2 and YY1 was not observed. We think the abundant oligo concentrations provided sufficient binding sites for all three factors, preventing competitive displacement. To further validate our model, we performed oligo4 titration assays comparing normal and YY1-depleted nuclear extracts as shown below. These experiments revealed clear competition thresholds: while no displacement occurs at 5μg or 1μg oligo conditions, reducing oligos to 0.2μg or 0.04μg demonstrates significant competitive effects (TFAP2 binding increases upon YY1 depletion when compare lane 5 to lane 9), confirming the competitive relationship under appropriate conditions. SE, short exporsure; LE, long exporsure.

TFAP2 knockdown in CaSki cells does not further increase lnc-FANCI-2 expression. In CaSki cells, we propose that endogenous YY1 levels are sufficiently high to occupy nearly all available competition sites at the lnc-FANCI-2 promoter (YY1-B motif). This speculation is supported by our previous work demonstrating that YY1 knockdown [3] or motif deletion[6] causes a 70–80% reduction in lnc-FANCI-2 expression in CaSki cells. These findings suggest a saturated promoter state in which lnc-FANCI-2 expression becomes fully dependent on YY1, while TFAP2 is already displaced from its key regulatory sites. This explains why further TFAP2 depletion fails to regulate the lnc-FANCI-2 expression in CaSki cells. We hypothesize that the competition between YY1 and TFAP2 is not highly dynamic but rather depends largely on the relative levels of YY1 and TFAP2 in different cell types, as illustrated in our model (Fig. 4).

TFAP2 shows similar overall promoter binding in both HFK and CaSki cells, whereas YY1 occupancy increases in CaSki cells, particularly peaking at the -308 to +108 region. Although the precise reason for this remains unclear, the resolution of our current ChIP method is not sufficient to determine the binding status at the specific competition sites (YY1 motifs). Notably, additional TFAP2A/2C binding sites were identified upstream of the YY1 competition site (Fig. S2D, Lane 3), suggesting that the overall TFAP2A/2C binding remains unchanged due to association with these alternative promoter regions. This residual binding may account for the observed pattern, but further experiments are required to validate this hypothesis. We have discussed this point in the manuscript: Lines 182-186 “Upon HPV infection, the upregulated YY1 protein competes with TFAP2, leading to the transactivation of lnc-FANCI-2. TFAP2 replacement occurs specifically at the YY1 motif B, while TFAP2 continues to bind other sites on the lnc-FANCI-2 promoter (Fig.3C and D). However, this residual TFAP2 binding does not appear to repress transcriptional activity.” We hope that our explanation, along with the additional Oligo4 titration assays, sufficiently addresses the reviewer's concern.

3. The authors developed a DNA oligo pull-down method to compare binding affinities or determine mutually exclusive binding (Fig. 1). However, the reliability of this new method is not further evaluated. It is recommended to include a positive control group or incorporate additional methods for comparative analysis to strengthen the validity of the findings.

Response: We appreciate the reviewer’s comment regarding the reliability of our DNA oligo pull-down method. To validate this assay, we incorporated an electrophoretic mobility shift assay (EMSA) to examine the binding interactions between TFAP2 proteins and oligo4 in the presence of exogenous YY1. As shown below (left), exogenous YY1 formed a distinct complex with oligo4, as indicated. The lower band likely represents a cleaved YY1 and DNA complex [7], which was also observed in the oligo pull-down assay (right). In addition, a complex was detected above the YY1–DNA complex, which we interpret as a potential TFAP2–DNA complex. This band was only observed in nuclear extracts with endogenous YY1 expression and was disrupted upon addition of exogenous YY1. These observations are consistent with the competitive binding model supported by the oligo pull-down results. Together, these data strengthen our conclusion that TFAP2 and YY1 bind to oligo4 in a mutually exclusive manner. We have revised the manuscript to include this supporting evidence for the oligo pull-down approach (Line 101-103, In addition, electrophoretic mobility shift assay (EMSA) further supported this competition model, as exogenous YY1 disrupted the potential TFAP2–oligo4 complex (Fig. S1C).

Reviewer #3: See above

# Part III – Minor Issues: Editorial and Data Presentation Modifications

Please use this section for editorial suggestions as well as relatively minor modifications of existing data that would enhance clarity.

Reviewer #1: N/A

Reviewer #2: 1. The writing needs to be improved to enhance the readability and logical flow of the manuscript. For example, the first two sentences of the Results section, 'YY1-B is more critical for the lnc-FANCI-2 promoter activity. To further identify the regulatory or binding proteins of the lnc-FANCI-2 core promoter, we performed DNA oligo pulldown-coupled mass spectrometry using soluble nuclear extract (Nuc) from HPV16+ CaSki cells with or without E7 knockdown,' are not clearly connected. The statement about YY1-B does not logically lead to the description of the experiment in the second sentence.

Response: We appreciate the reviewer’s constructive feedback. To address the concern about the connection between the two sentences in the Results section, we have revised the text to provide clearer context and logical progression. Specifically, we now introduce the role of YY1-B in lnc-FANCI-2 promoter activity earlier in the manuscript (both Introduction and Results) and explicitly link it to the rationale for identifying additional regulatory proteins. Line 48-53, “Two essential YY1 binding motifs upstream of lnc-FANCI-2's transcription start site are crucial for its transcriptional activity (Fig.1A), with YY1 knockdown or motif deletion causing a 70–80% reduction in lnc-FANCI-2 expression in cervical cancer cells. This study aims to identify additional host proteins binding to the lnc-FANCI-2 promoter through proteomic analysis and proposes a model for how TFAP2 and YY1 regulate lnc-FANCI-2 and other cancer-related genes in both primary and cancer cells.” Lines 57-62, “Although two YY1-binding motifs, YY1-A and YY1-B (Fig. 1A), in the lnc-FANCI-2 promoter region have very similar binding affinities to YY1, the YY1-B is more critical for the lnc-FANCI-2 promoter activity. To comprehensively characterize how HPV16 E7 modulates the transcriptional complex at this locus, we performed DNA oligo pull-down-coupled mass spectrometry using soluble nuclear extract (Nuc) from HPV16+ CaSki cells with or without E7 siRNA transfection (Fig.1A and B).”

2. The insertion of a working model in Figure 2 is not ideal for the structure of the manuscript. It would be better to introduce the relationships between YY1, TFAP2, E7, lnc-FANCI-2, and miR-29a in the Introduction and use Fig. 2 as an abstract diagram for clarity.

Response: We sincerely appreciate the reviewer's valuable suggestion regarding the presentation of our working model. We have revised the Introduction to explicitly introduce the key relationships between YY1, TFAP2, E7, lnc-FANCI-2, and miR-29a, providing essential context for readers before they encounter the experimental results. Specifically, we now describe how HPV16 E7 functionally interacts with YY1 to modulate transcriptional activity and explain the E7-miR-29a-YY1 regulatory axis involved in HPV-mediated gene expression changes. We have moved two supplementary figures to the main figures. To maintain the logical flow of the text, Fig. 2 has been relocated to Fig. 4, where it now serves as a clear schematic diagram.

In several Western blot experiments, p53 expression is measured (e.g., Fig. S1B, where E7 siRNA targeting both E6 and E7 transcripts resulted in the accumulation of nuclear p53). However, the role of p53 is not explained, and the connection between this result and the main theme of the article is unclear. Further clarification is needed on why p53 was measured and how these results tie into the overall hypothesis.

Response: We appreciate the reviewer’s astute observation regarding the inclusion of p53 data in our study. In the revised manuscript, we have clarified the rationale for monitoring p53 levels and its connection to our central hypothesis. In HPV-positive cervical cancer cells (like CaSki), E6 oncoprotein targets p53 for degradation. The accumulation of nuclear p53 upon E7 siRNA treatment (which also reduces E6 due to shared transcripts) confirms an effective E6/E7 knockdown. Lines 64-66: “Previous work demonstrated E7 siRNA also targeted E6 transcripts and resulted in the accumulation of nuclear p53 (Fig.1B and Fig.S1A)21. This effect was used as an indicator of effective E6/E7 knockdown. We identified 28 proteins in specific association with oligo 4 over the mutant oligo 4-M (>3 peptides with >2-fold changes), 13 of them related to knockdown-induced loss of binding (Table S1 and Fig.1C). “

Additionally, we have included a Western blot (Fig. S1) demonstrating the efficiency of E6 and E7 depletion with the E7-targeting siRNA in CaSki cells.

3. Since both YY1 and TFAP2 are transcription factors, their overlapping binding motifs likely affect the expression of numerous genes. In Figure 3B, the statement that "these CBS potentially regulate cell junction assembly, cell-cell adhesion via plasma membrane adhesion molecules, and keratinocyte proliferation" seems speculative without detailed reasoning or evidence linking these pathways directly to the mechanisms of HPV-induced carcinogenesis. The experiment design of genome-wide screening of YY1 and TFAP2 competitive binding sites to find genes related to HPV-driven carcinogenesis seems inadequately substantiated.

Response: We appreciate the reviewer’s valid critique regarding the interpretation of our functional enrichment analysis. In the revised manuscript, we have modified our conclusions to more accurately reflect the limitations of these bioinformatic predictions while maintaining their biological relevance to HPV-associated carcinogenesis. Lines 138-141: " Functional enrichment analysis suggested that these CBS potentially regulate carcinogenesis-related pathways, including cell junction assembly, cell-cell adhesion via plasma membrane adhesion molecules, and keratinocyte proliferation (Fig.5B and Table S3), although experimental validation is still required. "

4. Fig. 4A–B and Fig. S3 mention H3K27ac and H3K4Me3 marks in the promoter regions, but the manuscript briefly mentions these findings without explaining their significance or the impact they have on the study’s conclusions. More detail on these marks and their relevance to the research would be beneficial.

Response: We thank the reviewer for this important suggestion. In the revised manuscript, we explain the H3K27ac and H3K4me3 marks. Lines 148-149: “The presence of both H3K27ac (a marker of active enhancers) and H3K4me3 (a hallmark of active promoters) provides strong epigenetic evidence for the transcriptional activity of these loci.”

5. In Fig. 4G–H, the authors demonstrate that high levels of either PPP1R15B or LRRC37A correlate with a worse survival rate in cervical cancer patients. This experiment effectively supports the relevance of YY1 and TFAP2 competitive binding sites in cervical cancer and ties well into the manuscript’s central theme.

Response: We are particularly grateful for the reviewer's acknowledgment that these results effectively tie into our central theme of YY1/TFAP2-mediated transcriptional regulation in cervical carcinogenesis.

6. In the Discussion, the authors mention that "the residual TFAP2 binding does not repress transcription." This point is unclear and warrants further experimental verification or more in-depth discussion to clarify its implications.

Response: We appreciate the reviewer's valid concern regarding the interpretation of residual TFAP2 binding. We have revised the original statement about residual TFAP2 binding not repressing transcription. Lines 115-118, “Another specific TFAP2 binding event was identified at a distinct TFAP2 motif within the promoter region (Fig.3C–D, oligo1), which may explain the unchanged TFAP2 ChIP enrichment observed in CaSki cells”. Lines 182-184, “TFAP2 replacement occurs specifically at the YY1 motif B, while TFAP2 continues to bind other sites on the lnc-FANCI-2 promoter (Fig.3C and D). However, this residual TFAP2 binding does not appear to repress transcriptional activity.”

Reviewer #3: The text of the manuscript needs editing for correct English language use throughout.

Response: We appreciate the reviewer’s attention to language clarity. To ensure the manuscript meets high standards of English language usage, the manuscript has undergone language editing.

**References**

1.	Tang S, Tao M, McCoy JP, Zheng Z-M. The E7 oncoprotein is translated from spliced E6*I transcripts in high-risk human papillomavirus type 16- or type 18-positive cervical cancer cell lines via translation reinitiation. J Virol. 2006;80: 4249–4263. doi:10.1128/JVI.80.9.4249-4263.2006

2.	Stormo GD. Modeling the specificity of protein-DNA interactions. Quant Biol. 2013;1: 115–130. doi:10.1007/s40484-013-0012-4

3.	Liu H, Xu J, Yang Y, Wang X, Wu E, Majerciak V, et al. Oncogenic HPV promotes the expression of the long noncoding RNA lnc-FANCI-2 through E7 and YY1. Proc Natl Acad Sci U S A. 2021;118: e2014195118. doi:10.1073/pnas.2014195118

4.	Deng Z, Cao P, Wan MM, Sui G. Yin Yang 1: a multifaceted protein beyond a transcription factor. Transcription. 2010;1: 81–84. doi:10.4161/trns.1.2.12375

5.	Gordon S, Akopyan G, Garban H, Bonavida B. Transcription factor YY1: structure, function, and therapeutic implications in cancer biology. Oncogene. 2006;25: 1125–1142. doi:10.1038/sj.onc.1209080

6.	Liu H, Yu L, Majerciak V, Meyer T, Yi M, Johnson PF, et al. The long noncoding RNA lnc-FANCI-2 intrinsically restricts RAS signaling and phosphorylation of Akt and Erk in HPV16-infected cervical cancer. 2024. doi:10.7554/elife.102681.1

7.	Riman S, Rizkallah R, Kassardjian A, Alexander KE, Lüscher B, Hurt MM. Phosphorylation of the Transcription Factor YY1 by CK2α Prevents Cleavage by Caspase 7 during Apoptosis. Mol Cell Biol. 2012;32: 797–807. doi:10.1128/MCB.06466-11
