# Supplementary material for: A genome-wide analysis of YY1 and TFAP2 competition on overlapping motifs reveals their roles in HPV-induced carcinogenesis
Source: PLoS Pathog. 2025 Sep 15;21(9):e1013524. doi: 10.1371/journal.ppat.1013524 (PMC12445741; doi:10.1371/journal.ppat.1013524)
Supplement: Attachment — Submitted filename: Review comments on PPATHOGENS-D-25-00313 - R1.docx [file ppat.1013524.s011.docx]

Comments on "A genome-wide analysis of YY1 and TFAP2 competition on overlapping motifs reveals their roles in HPV-induced carcinogenesis" by Liu et al.

This report investigates the regulatory mechanism of the long non-coding RNA lnc-FANCI-2 in HPV-driven cervical carcinogenesis. While the manuscript provides novel insights into the antagonistic roles of YY1 and TFAP2 in regulating lnc-FANCI-2 and broader transcriptional networks, the manuscript has several shortcomings need to be addressed.

the writing lacks logical coherence, making it difficult for readers to follow the study’s flow. The rationale behind each experiment is not clearly articulated, nor is it easy to understand how specific conclusions are derived from the corresponding results. The manuscript would benefit greatly from improved organization and enhanced clarity to increase its readability.

Second, the logical framework of the manuscript is incomplete, particularly concerning the relationship between E7, YY1, and TFAP2. Although the authors express an intention to explore how E7 manipulates YY1 to drive HPV-induced transcriptional reprogramming, the manuscript lacks regulatory data directly linking E7 and YY1. This omission weakens the clarity and focus of the study's central objective.

Third, the biological relevance of the findings remains unsubstantiated. There are no experiments demonstrating that the proposed mechanism has a functional impact on HPV-induced tumorigenesis, which significantly diminishes the importance and credibility of the study.

Overall, this study provides compelling evidence of competitive binding between YY1 and TFAP2 at the lnc-FANCI-2 promoter through a series of well-executed experiments. With appropriate revisions to strengthen the logical and evidentiary framework of the manuscript, I would recommend this manuscript for acceptance.

Major Points:

1. The current study is limited to cell-based experiments. It is strongly recommended that the authors complement their findings with in vitro luciferase reporter assays to further validate the competitive binding of YY1 and TFAP2 to the lnc-FANCI-2 promoter.
2. In Figure 3E, the authors are encouraged to perform siYY1 in CaSki cells to determine whether TFAP2A binding increases upon YY1 knockdown and how this affects downstream lnc-FANCI-2 transcription. Conversely, YY1 overexpression in HFK cells should be conducted to assess whether TFAP2A binding to the lnc-FANCI-2 promoter is reduced and whether this impacts its transcription. These experiments would strengthen the mechanistic conclusions.

Minor Points:

1. The line and bar graphs throughout the manuscript do not include statistical significance analyses. Proper statistical validation is essential to support the conclusions drawn from these data.
