# Supplementary material for: A genome-wide analysis of YY1 and TFAP2 competition on overlapping motifs reveals their roles in HPV-induced carcinogenesis
Source: PLoS Pathog. 2025 Sep 15;21(9):e1013524. doi: 10.1371/journal.ppat.1013524 (PMC12445741; doi:10.1371/journal.ppat.1013524)
Supplement: Attachment — Submitted filename: minor revision.docx [file ppat.1013524.s012.docx]

**Reviewers' Comments:**

Reviewer's Responses to Questions

# Part I - Summary

Please use this section to discuss strengths/weaknesses of study, novelty/significance, general execution and scholarship.

Reviewer #1: The authors have correctly addressed all of my comments.

Reviewer #2: This report investigates the regulatory mechanism of the long non-coding RNA lnc-FANCI-2 in HPV-driven cervical carcinogenesis. While the manuscript provides novel insights into the antagonistic roles of YY1 and TFAP2 in regulating lnc-FANCI-2 and broader transcriptional networks, the manuscript has several shortcomings need to be addressed.

the writing lacks logical coherence, making it difficult for readers to follow the study’s flow. The rationale behind each experiment is not clearly articulated, nor is it easy to understand how specific conclusions are derived from the corresponding results. The manuscript would benefit greatly from improved organization and enhanced clarity to increase its readability.

Second, the logical framework of the manuscript is incomplete, particularly concerning the relationship between E7, YY1, and TFAP2. Although the authors express an intention to explore how E7 manipulates YY1 to drive HPV-induced transcriptional reprogramming, the manuscript lacks regulatory data directly linking E7 and YY1. This omission weakens the clarity and focus of the study's central objective.

Third, the biological relevance of the findings remains unsubstantiated. There are no experiments demonstrating that the proposed mechanism has a functional impact on HPV-induced tumorigenesis, which significantly diminishes the importance and credibility of the study.

Overall, this study provides compelling evidence of competitive binding between YY1 and TFAP2 at the lnc-FANCI-2 promoter through a series of well-executed experiments. With appropriate revisions to strengthen the logical and evidentiary framework of the manuscript, I would recommend this manuscript for acceptance.

**Response:** We sincerely thank Reviewer #2 for the thorough evaluation and constructive comments. We have undertaken revisions to enhance the logical flow and readability of the manuscript. Specifically, we have: 1) Revised the introduction to more clearly present the established E7-YY1-lnc-FANCI-2 axis as the foundational context for investigating additional regulatory mechanisms; 2) Added explicit rationales at the beginning of each results subsection that clearly state the scientific question being addressed and how the experimental approach will answer it. Revised the results narrative to directly connect specific findings to their corresponding conclusions, ensuring a clearer line of reasoning throughout (see lines 100-101, 108-112, and 116-120); and 3) Acknowledged the limitation that the functional impact of YY1/TFAP2 competition on tumorigenesis remains to be determined in the discussion. These revisions have substantially improved the logical coherence and overall readability of the manuscript.

Based on our previous findings, we established a direct mechanistic link between the HPV16 oncoprotein E7 and the host transcription factor YY1 that is central to the activation of lnc-FANCI-2 [1]. We demonstrated that HPV16 E7 physically interacts with YY1 in a DNA-independent manner, with the CR3 domain of E7 being critical for this binding. This interaction is functionally significant, as knockdown of either E7 or YY1 significantly impairs lnc-FANCI-2 promoter activity. Furthermore, we showed that E7 promotes the transactivator function of YY1 on this promoter, a role that is independent of YY1's acetylation status or its potential interaction with the acetyltransferase p300. This established E7-YY1 axis provides the foundational mechanism for the transcriptional upregulation of lnc-FANCI-2 upon HPV infection. A paragraph detailing the functional interaction between HPV16 E7 and YY1, and its role in lnc-FANCI-2 transactivation, has been incorporated into the revised introduction (lines 51–54) to provide essential mechanistic context for this study.

We agree with the reviewer that demonstrating the functional impact of the YY1/TFAP2 competition on tumorigenesis represents an important direction for future research. While the current study focuses on elucidating the mechanistic basis of this transcriptional competition, we acknowledge that establishing direct links to tumorigenic phenotypes requires further investigation in more complex model systems. We have explicitly addressed this point in the discussion section (lines 215-221), where we propose plans for future studies utilizing HPV-positive organoid models and patient-derived xenografts to functionally validate whether the competitive displacement of TFAP2 by YY1 directly drives oncogenic phenotypes such as sustained proliferation, invasion, and in vivo tumor growth.

Reviewer #3: This is a resubmitted manuscript. The authors have taken on board all of my suggestions from the first round of reviews and I think the manuscript is much improved.

# Part II – Major Issues: Key Experiments Required for Acceptance

Please use this section to detail the key new experiments or modifications of existing experiments that should be absolutely required to validate study conclusions.

Generally, there should be no more than 3 such required experiments or major modifications for a "Major Revision" recommendation. If more than 3 experiments are necessary to validate the study conclusions, then you are encouraged to recommend "Reject".

Reviewer #1: N/A

Reviewer #2: 1. The current study is limited to cell-based experiments. It is strongly recommended that the authors complement their findings with in vitro luciferase reporter assays to further validate the competitive binding of YY1 and TFAP2 to the lnc-FANCI-2 promoter.

Response: We appreciate the reviewer’s comment. We have performed the in vitro luciferase reporter assays in our previously published study [1,2]. As shown in Fig. 5 of reference 1 (see figure below), we observed that the promoter activity of lnc-FANCI-2 in CaSki cells was regulated by YY1, consistent with the reduction of lnc-FANCI-2 expression upon YY1 knockdown. Successive 5′-to-3′ deletion and mutation assays demonstrated that the promoter activity was driven by regions containing YY1 binding motifs A and B, with motif B identified as a competitive binding site for TFAP2 and YY1. These findings indicate that lnc-FANCI-2 expression in CaSki cells is strongly dependent on YY1 binding under conditions of high YY1 expression.

In addition, we performed in vitro luciferase reporter assays under YY1 overexpression, which strongly repressed promoter activity (blue bars). When YY1 was first depleted by siRNA, YY1 overexpression instead enhanced promoter activity (see figure below; partial data published as Fig. S7 in reference 1). These results suggest that an appropriate level of YY1 is required for promoter activation, whereas excessive YY1 may act as a negative regulator by titrating essential components of the transcriptional machinery.

However, when we performed in vitro luciferase reporter assays under TFAP2 knockdown (see figure below, unpublished data), promoter activity was reduced even when the competitive binding site B was mutated, indicating that TFAP2 regulates promoter activity through additional mechanisms. For example, TFAP2 may act via other motifs beyond site B, as another specific TFAP2 binding event was identified within the promoter region (Fig. 3C–D, oligo1). These findings also highlight that the luciferase assay cannot fully recapitulate the chromatin context.

2. In Figure 3E, the authors are encouraged to perform siYY1 in CaSki cells to determine whether TFAP2A binding increases upon YY1 knockdown and how this affects downstream lnc-FANCI-2 transcription. Conversely, YY1 overexpression in HFK cells should be conducted to assess whether TFAP2A binding to the lnc-FANCI-2 promoter is reduced and whether this impacts its transcription. These experiments would strengthen the mechanistic conclusions.

Response: We thank the reviewer for this thoughtful suggestion. We agree that examining the dynamics of TFAP2 and YY1 binding under different conditions would further strengthen the mechanistic understanding. The following oligo pull-down assays using nuclear extracts from control and YY1-depleted CaSki cells might address this concern. While no displacement was observed under high oligo conditions (5 μg or 1 μg), reducing the oligo amount to 0.2 μg or 0.04 μg resulted in significant TFAP2 enrichment upon YY1 depletion (lane 5 vs. lane 9), demonstrating the competitive relationship under appropriate thresholds. These data are included in Fig. S1D. This experiment, conducted under strictly controlled conditions, removes all influences of the chromatin environment and directly demonstrates that the competition between YY1 and TFAP2 for the same DNA sequence is intrinsic and direct. This provides fundamental mechanistic evidence supporting their competitive relationship in cellular contexts.

Regarding the reviewer’s suggestion to test YY1 overexpression in HFK cells, we regret that this experiment cannot be performed at present because the primary HFK cells originally used in our study are no longer available. Our laboratory is still in the process of re-establishing protocols and obtaining primary HFK cells, which currently prevents us from performing YY1 overexpression experiments.

Reviewer #3: On reading the manuscript again, I now note that the ChIP experiments presented in Fig 3 are actually end point PCR and the data obtained from densitometry of PCR products separated by electrophoresis. I do not find the gel images anywhere in the manuscript. Perhaps these should be submitted as supplementary figures? Perhaps more worrying is that analysis of ChIP by end point PCR/densitometry is notoriously inaccurate. qPCR is a much better method for quantifying ChIP experiments and I suggest the authors consider reanalyzing their data in this way before the manuscript is accepted for publication.

Response: We thank the reviewer for the insightful comment regarding the use of qPCR for ChIP quantification. We agree that qPCR is a recognized gold standard for quantitative analysis.

The ChIP experiments in question were performed several years ago during the initial discovery phase of this project, at a time when endpoint PCR followed by densitometric analysis was the standard approach in our laboratory. These experiments were conducted under the supervision of Dr. Zhi-Ming Zheng. Our newly established laboratory is still in the process of re-establishing protocols and regaining access to the specific reagents and rare biological materials (including primary HFK cells) that were originally used. Unfortunately, this prevents us from immediately repeating the ChIP experiments using qPCR in the current revision cycle.

To ensure transparency and rigor, we now provide the full, uncropped gel images of all endpoint PCR results below and croped images in Fig.3A. Although endpoint PCR is not equivalent to qPCR, these data clearly and qualitatively demonstrate the key binding events that support our mechanistic model. We believe the current dataset is sufficient to substantiate the main mechanistic conclusions of this study, while recognizing that more quantitative analyses could further refine these findings in future investigations.

**Part III – Minor Issues: Editorial and Data Presentation Modifications**

Please use this section for editorial suggestions as well as relatively minor modifications of existing data that would enhance clarity.

Reviewer #1: N/A

Reviewer #2: 1. The line and bar graphs throughout the manuscript do not include statistical significance analyses. Proper statistical validation is essential to support the conclusions drawn from these data.

Response: We appreciate the reviewer’s suggestion. Statistical analyses have been performed and are included in Figs. 3A and 3E. For the line graphs derived from representative western blot quantifications, statistical testing was not applied, as these data were intended to illustrate band intensity changes from single representative experiments rather than from biological replicates.

Reviewer #3: (No Response)

References

1.	Liu H, Xu J, Yang Y, Wang X, Wu E, Majerciak V, et al. Oncogenic HPV promotes the expression of the long noncoding RNA lnc-FANCI-2 through E7 and YY1. Proc Natl Acad Sci U S A. 2021;118: e2014195118. doi:10.1073/pnas.2014195118

2.	Liu H, Yu L, Majerciak V, Meyer T, Yi M, Johnson PF, et al. The long noncoding RNA lnc-FANCI-2 intrinsically restricts RAS signaling and phosphorylation of Akt and Erk in HPV16-infected cervical cancer. 2024. doi:10.7554/elife.102681.1
